# Induction of colistin resistance and environmental toxicity assessment in *Escherichia coli*

**DOI:** 10.1371/journal.pone.0340467

**Published:** 2026-04-21

**Authors:** Thalita Hellen Nunes Lima, Mariana de Souza, Jennifer Machado Soares, Kate Cristina Blanco, e Vanderlei Salvador Bagnato

**Affiliations:** 1 São Carlos Institute of Physics, University of São Paulo, São Carlos, Brazil; 2 Biomedical Engineering, Texas A&M University, College Station, Texas, United States of America; Yamagata University Faculty of Medicine: Yamagata Daigaku Igakubu Daigakuin Igakukei Kenkyuka, JAPAN

## Abstract

The rise of Antimicrobial Resistance (AMR) among diverse microbial groups presents a growing threat to global public health. Colistin has become a last-line therapeutic option against multidrug-resistant Enterobacteriaceae. However, the global dissemination of mobile colistin resistance (mcr) genes, particularly in *Escherichia coli*, has raised significant concern. Among Gram-negatives, *E. coli* harbors the greatest diversity of *mcr* variants (mcr-1 to mcr-5), underscoring its importance as a model organism for resistance studies. This study investigated whether repeated exposure to subinhibitory concentrations of colistin sulfate could induce resistance in *E. coli* strain ATCC 25922. The strain was subjected to 10 growth cycles with 1.1 mg·L ⁻ ¹ of colistin, followed by one cycle at 6.4 mg·L ⁻ ¹. Minimum inhibitory concentrations (MICs) for colistin, ceftazidime, and gentamicin were determined, along with resistance maintenance assays and phenotypic evaluation of *mcr-1* gene expression. All results were compared to a clinically isolated multidrug-resistant *E. coli* strain (CCBH 20178). Exposure to subinhibitory doses of colistin successfully induced resistance, with increased MIC values observed for colistin from 2 to 16mgL^-1^. Although resistance to ceftazidime and gentamicin was not established, a moderate elevation in ceftazidime MIC was noted, even after antibiotic withdrawal. Phenotypic expression of *mcr-1* was detected in both the induced and clinical strains. Especially, the induced strain exhibited a two-fold increase in growth rate compared to susceptible control. These findings demonstrate that low-dose colistin exposure can promote the emergence of resistant *E. coli* strains and suggest a potential link between subinhibitory antimicrobial pressure and *mcr-1*-mediated resistance.

## Introduction

AMR among various microbial classes continues to escalate as a significant global health threat in the 21st century [1,2]. Gram-negative bacteria, particularly *Enterobacteriaceae* [3], are of special clinical concern due to their association with severe infections and their ability to acquire multidrug resistance. Colistin, also known as polymyxin E, is a cationic antimicrobial peptide produced by *Paenibacillus polymyxa* [4] and is currently considered a last-line therapeutic agent for infections caused by carbapenem-resistant Gram-negative pathogens [4,5].

Although colistin is a last-resort antimicrobial for treating Gram-negative bacterial infections [5], recent decades have seen a growing emergence of colistin-resistant Gram-negative strains [6–8]. Horizontal gene transfer of *mcr*-mediated (mobile colistin resistance) has been documented worldwide, representing a critical emerging threat to global health systems [9,10]. Currently, nine *mcr* allelic variants are known in scientific literature (*mcr*-1, 2, 3, 4, 5, 6, 7, 8, 9) [9]. *Escherichia coli* carries the most diverse *mcr* repertoire among Gram-negatives, with five identified variants (*mcr-1* through *mcr-5*) [9].

Horizontal gene transfer facilitates the spread of resistance by transmitting resistance genes to neighboring microorganisms via plasmids, rather than being limited to direct descendants [6]. Without new alternatives, spreading resistance to last-resort antimicrobials like colistin will lead to increased treatment failures and higher mortality rates in Gram-negative bacterial infections [10]. The rise of colistin-resistant *E. coli* strains has been widely reported in human clinical and animal husbandry settings worldwide, signaling a critical One Health issue [8–10].

Microorganisms that develop colistin resistance frequently exhibit resistance to other antimicrobial classes, making them multidrug-resistant organisms [11]. Notably, as of 2020, the *mcr*-1 gene remains the most widely reported variant globally, having been detected in 61 countries across all inhabited continents [12]. Following the initial discovery of the *mcr-*1 gene in 2016, nine additional *mcr* variants were identified within just three years [9]. The rapid global spread of *mcr* variants demands immediate research into Colistin resistance evolution and mitigation. Furthermore, the availability of colistin in ecosystems is a concern both in the context of antimicrobial resistance dissemination and in the scenario of environmental toxicity. *Allium cepa* is a bioindicator plant species used in various ecotoxicological studies investigating the effects of contaminants in different environmental matrices [13].

This study will evaluate resistance selection in *E. coli* under sub-MIC colistin pressure and assess collateral resistance to antibiotics like cephalosporins, β-lactams, and aminoglycosides. This study will evaluate whether sub-MIC concentrations of colistin can alter the phenotypic traits of *E. coli.* Furthermore, *A. cepa* will be used as a bioindicator organism to assess the environmental toxicity of subinhibitory and inhibitory concentrations of Colistin.

## Materials and methods

### Determination of Initial Minimum Inhibitory Concentration (MIC)

*E. coli* ATCC 25922 strains were maintained in Brain Heart Infusion (BHI) broth supplemented with 20% glycerol and stored at −20°C. For experimental use, strains were streaked onto sterile BHI agar plates using the quadrant streak method and incubated at 37°C for 24 hours.

After incubation, isolated colonies were suspended in 10 mL of phosphate-buffered saline (PBS, pH 7.4) and homogenized by vortexing. Commercial antimicrobial agents – (i) Colistin sulfate (Sigma-Aldrich), (ii) Gentamicin (Sigma-Aldrich), and (iii) Ceftazidime (Sigma-Aldrich) – prepared by serial microdilution. Ceftazidime and gentamicin were diluted in Mueller-Hinton (MH) broth, while colistin sulfate was diluted in cation-adjusted Mueller-Hinton broth (CAMHB). The MIC values for all antimicrobials were determined according to BrCAST guidelines [14]. Bacterial suspensions (5 × 10^5^ CFU/mL) were inoculated into each well containing the antimicrobial dilutions, followed by the addition of 0,015% (w/v) resazurin solution (Sigma-Aldrich). Metabolic activity was assessed after 4 hours of incubation at 37 °C. The subinhibitory concentration, corresponding to 40% of the MIC, was selected for subsequent resistance induction experiments.

### Antimicrobial resistance induced experiments

The *E. coli* (ATCC 25922) strain was maintained in Brain Heart Infusion (BHI) broth supplemented with 20% glycerol and stored at −20°C. For experimental procedures, 10 µL of the cryopreserved culture was inoculated into 10 mL of liquid BHI and incubated at 37 °C for 24 hours.

Following incubation, cultures were divided into two groups: the treatment group was exposed to 1.1 mg·L ⁻ ¹ of colistin sulfate (clinically relevant subinhibitory concentration), while the control group remained antibiotic-free under identical conditions. The antimicrobial resistance induction protocol was adapted from Ching *et al.,* (2020) [15].

Cultures were subjected to ten consecutive 24-hour growth cycles, with complete medium replacement containing fresh colistin after each cycle. MIC determinations were performed after every even-numbered cycle (C2, C4, C6, C8, and C10) using the standard broth microdilution method. After completing these ten selection cycles, the cultures underwent an additional validation cycle (C11) where they were exposed to antimicrobial at a concentration equivalent to 40% of the C10-determined MIC (specifically 6.4 mg/L in this experimental series). This final 24-hour exposure was immediately followed by conclusive MIC testing to characterize the acquired resistance phenotype.

The resulting induced strain, demonstrating stable resistance after this serial passage protocol, was formally designated as *E. coli* (C11) for subsequent investigations. Throughout the entire procedure, all cultures were maintained under controlled environmental conditions 37°C with triplicate biological replicates to ensure methodological rigor.

### Resistance maintenance experiments

The *E. coli* strains (ATCC 25922, CCBH 20178, and induced resistant variant C11) were maintained in Brain Heart Infusion (BHI) broth supplemented with 20% glycerol and stored at −80°C. For experimental procedures, 10 μL aliquots of frozen stocks were inoculated into 10 mL of fresh BHI broth and incubated at 37°C for 24 hours under controlled conditions.

Subsequently, bacterial cultures underwent six consecutive 24-hours growth cycles in liquid BHI medium, with complete medium replacement after each cycle. Following each passage growth cycle, MIC determinations were performed for three antimicrobial agents: (i) colistin sulfate (Sigma-Aldrich), (ii) gentamicin (Sigma-Aldrich) as an aminoglycoside, and (iii) ceftazidime (Sigma-Aldrich) as a cephalosporin and β-lactam. The MIC were performed using the standardized broth microdilution method described previously. This protocol allowed the monitoring of resistance stability and susceptibility profiles throughout the experimental period. The experiments were performed in triplicate. The data showed no parametric distribution, so for the statistical analyses, Kruskal-Wallis ANOVA was used, with a 95% significance interval (P = 0.05).

### Growth analysis

*E. coli* strains (ATCC 25922, CCBH 20178, and the induced resistant variant (C11) were maintained in Brain Heart Infusion (BHI) broth supplemented with 20% glycerol and stored at −20°C. For growth assays, strains were streaked onto sterile BHI agar plates using the quadrant streaking method and incubated at 37°C for 24 hours. Selected colonies were suspended in 10 mL of phosphate-buffered saline (PBS, pH 7.4) and homogenized by vortexing. The bacterial suspension was diluted to an optical density of 0.13 at 600 nm (OD_600nm_), followed by a 1:100 dilution in sterile PBS. For growth curve analysis, 200 µL of fresh BHI broth was distributed into each well of a 96-well microplate, followed by inoculation with 50 µL of the diluted bacterial suspension. The plate was incubated in a microplate reader at 37°C with continuous shaking. Bacterial growth was monitored by measuring OD_600nm_ at 15-minute intervals over 12-hour period. The generation time was determined from the slope of the curve during the logarithmic growth phase and using Equation 1. As the data followed a normal distribution, the Student’s t-test was performed.


$$Generation\;time=\;\frac{\text{ln}(2)}{{growth\;rate}_{logarithmical\;phase}}$$ (1)

### Phenotypic evaluation of mcr-1 gene on *E. coli* strains

Following the eleven-cycle induction protocol with colistin sulphate, phenotypic expression of the *mcr-1* gene was evaluated using the methodology described by Gwozdzinski et al. (2018) [16]. This protocol enables the identification of *mcr-1*-mediated colistin resistance based on differential MIC values obtained in calcium-supplemented culture media. The evaluation was based on MIC determinations performed under two distinct conditions: (i) conventional testing using standard CAMHB, and (ii) modified testing using CAMHB supplemented with 5 mM calcium chloride dehydrate. The calcium-enriched medium was prepared aseptically by adding a filter-sterilized 0.5 M solution of calcium chloride dehydrate (Êxodo Química) to CAMHB under sterile conditions. This proof-of-concept approach relies on the biochemical mechanism by which *mcr-1*-encoded phosphoethanolamine transferase activity increases bacterial resistance to colistin, reflected by elevated MIC values in the presence of calcium ions [16].

### *Allium cepa* test

For the environmental toxicity tests, seeds of *A. cepa* (Baia Periforme var.) free of agrochemicals (Isla Sementes Ltda., Brazil) were used, distributed into 4 groups (n = 90 per group). The experiments were conducted in triplicates. The seeds were germinated at 23 ± 2°C under a 12:12 h photoperiod and 60 ± 10% relative humidity for 96 hours. Each group was irrigated daily with different chlorine concentrations (1.1, 6.4, 12.8 mg.L^-1^) while the control group received only distilled water. After 96 hours, the seeds were collected, measured, counted, and stored until slide preparation. The germination index (GI) was used to quantify phytotoxicity, calculated after 96 h (Equation 2). The germinated seeds were measured with a digital caliper (Digimess 100.178/200 mm) to determine the growth index and analyzed according to Equation 3.


$$GI=\frac{Total\;of\;germinated\;seeds}{Total\;mumber\;of\;seeds}\;\times 100$$ (2)

$$REI=\frac{Sum\;of\;rootlets^{{\;}^{\prime }}length}{Toral\;of\;rootlets}$$ (3)

The experiments were performed in triplicate (N = 90). The data showed a normal distribution, so for the statistical analyses, one-way ANOVA was used, with a 95% significance interval (P = 0.05). Tukey#39;s post-hoc test further evaluated groups that showed significant differences between means

### Statistical analysis

For all obtained data, normality tests were performed to determine the appropriate statistical analysis. All statistical analyses were conducted using Origin 2024 software (OriginLab®).

## Results

### Initial MIC results

The MIC for *E. coli* (ATCC 25922) was 2 mg·L ⁻ ¹ for Colistin, 0.125 mg·L ⁻ ¹ for ceftazidime, and 1 mg·L ⁻ ¹ for gentamicin. According to the breakpoints established by BrCAST for colistin sulphate [17], ceftazidime [18], and gentamicin [18], *E. coli* (ATCC 25922) shows no resistance to these antimicrobials. Furthermore, the ATCC 25922 strain is commonly used as a standard non-resistant organism for evaluating colistin resistance [17]. In contrast, the *E. coli* (CCBH 20178) strain was resistant to all tested antibiotics, with MIC values of 8 mg·L ⁻ ¹ for colistin, 16 mg·L ⁻ ¹ for ceftazidime, and 64 mg·L ⁻ ¹ for gentamicin.

### Induced antimicrobial resistance results

**Fig 1:**
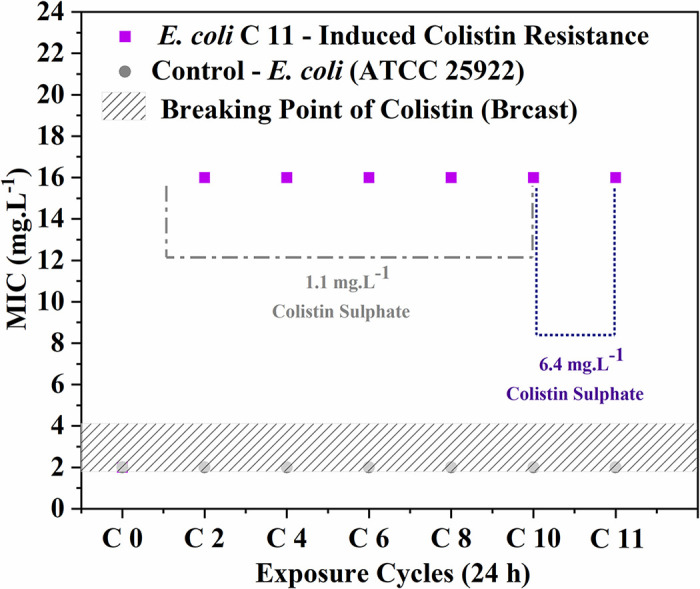
MIC of *E. coli* during sub-MIC concentrations exposed. The results illustrate the MIC progression of bacterial strains exposed to 1.1 mg.L^-1^ colistin over 10 consecutive cycles, followed by an eleventh cycle (C11) at an elevated concentration of 6.4 mg.L^-1^. The purple squares represent the MIC values of the strain undergoing subinhibitory colistin exposure, while the gray circles denote the control strain#39;s MIC values. The hatched square indicates the clinical breakpoint range of 2-4 mg/L, as established by BrCAST guidelines [13,21].

Fig 1 demonstrates that colistin resistance first becomes detectable after the second exposure cycle and remains stable throughout all 11 growth cycles. This graphical representation clearly shows the progressive development of resistance in the test strain compared to the stable susceptibility profile of the control, with the induced strain ultimately exceeding the clinical resistance threshold by the final cycles.

The MIC of *E. coli* (C11) to colistin demonstrated an 8-fold increase following consecutive exposure to subinhibitory doses of colistin sulphate. After 2 growth cycles at 1.1 mg.L^-1^ of colistin sulphate, the strain reached a final MIC of 16 mg.L^-1^, representing an approximate 14.5-fold increase. This elevated MIC was consistently maintained throughout all 10 cycles. Notably, when challenged with an elevated concentration of 6.4 mg.L^-1^ during the eleventh cycle (C11) of induction, the MIC remained stable at 16 mg.L^-1^, indicating the establishment of a robust resistance phenotype. The MIC increase observed in strain C11 (up to 16 mg·L ⁻ ¹) places it well beyond clinical resistance thresholds, possibly rendering colistin therapeutically ineffective against such adapted phenotypes.

**Fig 2:**
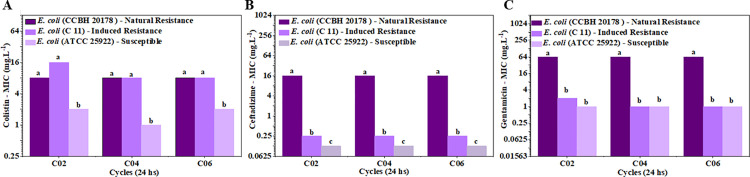
MIC profiles of *Escherichia coli* strains across six consecutive antibiotic-free growth cycles. **(A)** MIC for colistin; **(B)** MIC for ceftazidime; **(C)** MIC for gentamicin. Strains evaluated: *E. coli* ATCC 25922 (susceptible control – blue triangles), *E. coli* C11 (colistin-induced resistant strain – light purple circles), *E. coli* CCBH 20178 (clinical multidrug-resistant isolate – dark purple squares). Data represents the mean values of three independent biological replicates. Different letters indicate statistically significant differences, as determined by Kruskal-Wallis ANOVA (p < 0.05).

Fig 2 shows the resistance stability monitoring data across six subsequent growth cycles conducted in antimicrobial-free medium, revealing the persistence of the acquired resistance in the absence of selective pressure. We were interested in seeing if culturing with colistin at a sub-inhibitory concentration also affected sensitivity of other antimicrobial classes, including aminoglycosides and polymyxins.

Although the induced resistant strain *E. coli* (C11) did not develop complete resistance to gentamicin or ceftazidime, its MIC values doubled for ceftazidime, compared to the reference strain *E. coli* (ATCC 25922). The MIC values of ceftazidime for *E. coli* (C11) show a statistically significant difference when compared with *E. coli* (ATCC 25922). This moderate but consistent increase in MIC suggests potential low-level cross-resistance development or adaptive changes in membrane permeability during colistin resistance induction.

**Fig 3:**
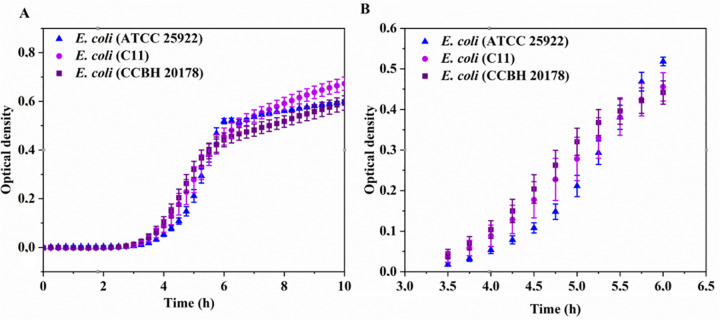
Microorganisms growth curve. (**A)**: Bacterial growth curve for the *E. coli* strains (ATCC 25922), *E. coli* (C 11), and the *E. coli* strain (CCBH20178). (**B)**: Bacterial growth curve of de logarithmical phase for the *E. coli* strains (ATCC 25922), *E. coli* (C 11), and the *E. coli* strain (CCBH20178).

Fig 3 presents comparative growth curve, for all three strains: (i) the susceptible control (*E. coli* ATCC 25922), (ii) the colistin-induced variant (*E. coli* C11), and (iii) the clinical isolate (*E. coli* CCBH 20178).

The colistin-induced resistant strain *E. coli* (C11) – derived from *E. coli* (ATCC 25922) – demonstrated enhanced growth compared to its non-induced parent strain. Table 1 shows the growth rate during the logarithmic phase and the doubling time.

**Table 1:** Growth rate and duplication time of *E. coli* strain.

Strain	Growth rate (h^-1^)	Duplication time (min)
***E. coli*** **(ATCC 25922)**	1,2 ± 0,1^a^	35 ± 3^a^
***E. coli*** **(C 11)**	2,3 ± 0,3^b^	19 ± 1^b^
***E. coli*** **CCBH (20178)**	2,1 ± 0,1^b^	20 ± 2^b^

Results of growth rates and duplication time of *E. coli* (ATCC 25922), *E. coli* (C 11) and *E. coli* (CCBH 20178) obtain based on growth rates. Different letters indicate statistically significant differences, as determined by the Student’s t-test (p < 0.05).

The clinical isolate *E. coli* (CCBH 20178) and the laboratory-induced resistant strain *E. coli* (C11) exhibited comparable growth rate, suggesting similar metabolic activity between naturally occurring and experimentally induced resistant phenotypes.

### Phenotypic evaluation of the *E. coli* strains and environmental toxicity analysis

**Fig 4:**
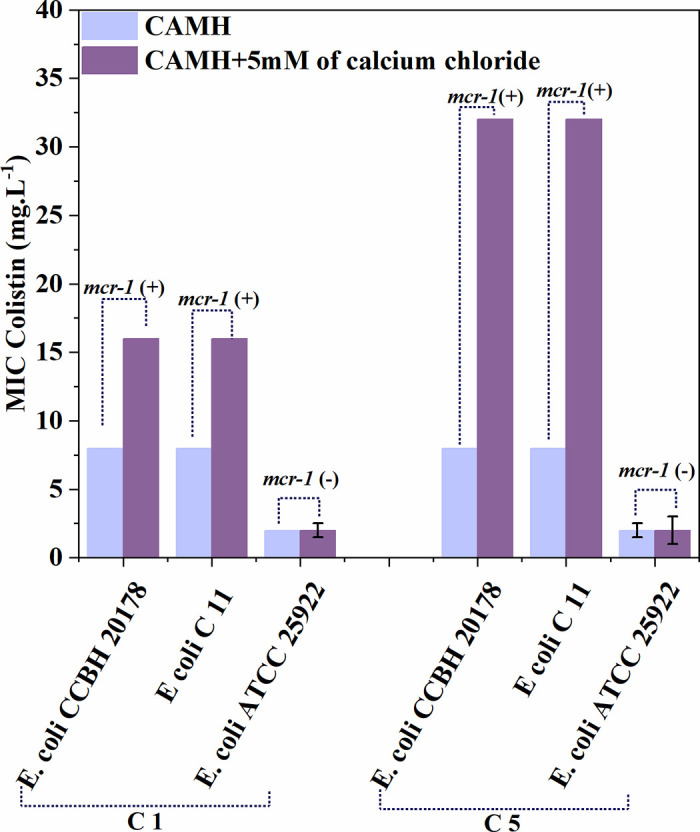
Phenotypic evaluation of the *E. coli* strains. The colistin sulfate MIC results in MHCA (cation-adjusted Mueller-Hinton broth, blue bars) and calcium chloride-enriched MHCA (5 mM) (purple bars) for *E. coli* strains (ATCC 25922), *E. coli* (C 11), and *E. coli* (CCBH 20178). Group C1 represents strains after one antimicrobial-free growth cycle, while group C5 shows results after five antibiotic-free growth cycles. Phenotypic detection of *mcr-1* was inferred from the MIC profiles and annotated above the bars as *mcr*-1(+) or *mcr*-1(–).

Fig 4 presents the results of the phenotypic evaluation of the *E. coli* strains, including *E. coli* (CCBH 20178) as the positive control, *E. coli* (C11), which is the strain obtained after resistance induction, and *E. coli* (ATCC 25922).

**Fig 5:**
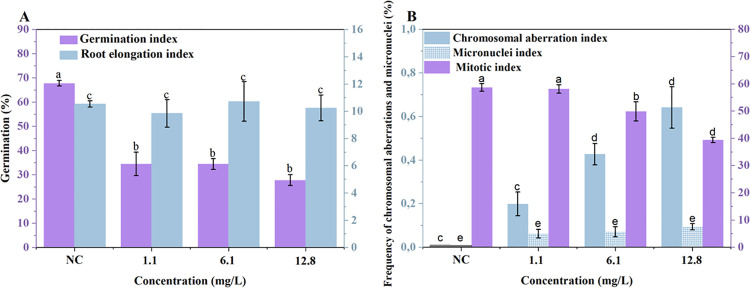
Environmental toxicity of colistin. **(A)** Macroscopic parameters of *A. cepa* seed germination and early development exposed to different concentrations of colistin, represented by the germination index and root elongation index. **(B)** Microscopic parameters, including the chromosomal aberration index, micronucleus index, and mitotic index. Data are presented as mean ± standard error (SE). Different letters above the bars indicate significant differences between treatments according to one-way ANOVA followed by Tukey’s post hoc test (p < 0.05).

*E. coli* (ATCC 25822) did not exhibit phenotypic expression of the *mcr*-1 gene, whereas the *E. coli* (CCBH 20178) strain showed a positive phenotype. Results demonstrated that in the first growth cycle followed by the completion of resistance induction, the induced strain (*E. coli* C 11) displayed phenotypic expression of the *mcr-1* gene. Furthermore, the induced strain maintained a positive *mcr-1* phenotype after five antimicrobial-free growth cycles. To determine whether subinhibitory concentrations of *E. coli* induce bacterial resistance to antibiotics (Fig 1) and cause environmental toxicity, the *A. cepa* test was performed. Fig 5 shows the environmental, genotoxic, and mutagenic analyses of colistin in *A. cepa.*

The 33% average reduction in germination rate suggests that colistin may be environmentally toxic. The results indicate that exposure to colistin affects cell division by decreasing the mitotic index at 6.4 mg.L^-1^ and 12.8 mg.L^-1^, respectively. In addition, the increase in the occurrence of chromosomal aberrations and micronuclei were also observed with statistical differences for the concentrations 6.4 mg.L^-1^ and 12.8 mg.L^-1^. There was no statistical difference between the 1.1 mg.L^-1^ concentration and the control group for the evaluated parameters.

## Discussion

Despite the environmental toxicity that our results presented, Colistin sulphate is an antimicrobial intended to treat infections caused by gram-negative strains [5]. The complete mechanism of action of colistin sulphate remain to be fully elucidated, and its bactericidal effects are primarily mediated through ionic interactions with the anionic lipid A component of lipopolysaccharide (LPS) in the outer membrane of Gram-negative microorganisms [4,10,19]. The model for the mechanism of action established to date is that colistin is electrostatically attracted to the anionic region of lipid A of lipopolysaccharide in the outer membrane [19]. Following initial binding, colistin displaces Mg²⁺ and Ca² ⁺ ions that usually stabilize the lipopolysaccharide (LPS) layer [4,19]. The biological membrane becomes destabilized through this mechanism, allowing intracellular components to leak into the extracellular environment [4].

Our results demonstrate that colistin sulphate resistance first emerges during the second growth cycle and remains stable throughout all eleven exposure cycles. Notably, the induced strain maintained this resistant phenotype even after six subsequent growth cycles in antibiotic-free medium. This persistence of resistance following removal of selective pressure indicates that the observed colistin resistance represents a permanent adaptation rather than a transient physiological response [20]. The strain exposed to sublethal doses of colistin sulphate did not develop resistance to ceftazidime and gentamicin. However, an increase in the MIC values for ceftazidime was observed, suggesting that the induction of colistin sulphate resistance reduced the inactivation efficiency of this antibiotic was assessed based on the significant difference between *E. coli* (C11) and its parental strain.

The study demonstrated phenotypic expression of the *mcr-1* resistance gene in both the experimentally induced strain and the positive control strain (*E. coli* CCBH 20178), a known carrier of *mcr-1*-mediated colistin resistance. This observation confirms that the resistance induction protocol successfully generated a strain exhibiting the characteristic *mcr-1* phenotype, with expression patterns equivalent to those observed in naturally occurring resistant clinical isolates. The parallel behavior between the induced and control strains provides strong evidence that the acquired resistance mechanism mirrors the well-characterized *mcr-1* pathway, including its typical lipid A modification profile and calcium-dependent resistance patterns [11]*.* The presence of the *mcr-1* gene is also related to virulence factors, growth rates [21], changes in the morphology [22] and fluidity of the biological membrane of microorganisms [23], and changes in the charge of the outer membrane [23,24]. Although the phenotypic expression of the *mcr-1* gene was confirmed, the gradual profile of resistance acquisition suggests that other mechanisms, such as mutations in *mgrB* or regulation of the *pmrAB* and *phoPQ* pathways, may also be involved in the resistant phenotype observed in Fig 1. These findings may be directly associated with the observed increases in MIC values for ceftazidime and the enhanced bacterial growth rates documented in our study. The concurrent emergence of these phenotypic alterations suggests a potential mechanistic link between *mcr-1*-mediated membrane modifications and the development of collateral resistance to structurally unrelated antimicrobial agents.

Our results demonstrate significant effects of colistin sulfate resistance on the bacterial growth rate during the logarithmic phase. Following resistance induction, the bacterial growth rate is different compared to the susceptible parental strain. Notably, the induced resistant strain exhibited growth rate similar to the naturally resistant clinical isolate.

Our phenotypic assessments, MIC determinations, and antibiotic-free growth experiments demonstrate that repeated sub-inhibitory exposure to colistin sulphate induces stable, heritable resistance. Three clinically significant effects accompanied this resistance development: collateral decreased susceptibility, enhanced growth dynamics, and genetic and phenotypic changes. Based on our results, we can suggest that the resistance mechanism may confer a selective growth advantage and membrane modifications. In addition to the observed antimicrobial resistance, we also evaluated the effects of continuous exposure to colistin sulphate on plant organisms.

The continuous exposure to Colistin Sulphate demonstrated toxic potential for *A. cepa*, and all tested concentrations showed a significant reduction in germination rate. However, the observed toxicity in the germination index did not significantly affect root growth in *A. cepa*. Exposure to colistin sulphate negatively affected cell division at concentrations of 6.4 and 12.8 mg·L ⁻ ¹, evidenced by a significant decrease in the Mitotic Index (MI) and a significant increase in the Nuclear Abnormality Index (NAI). Additionally, a non-significant increase in Micronucleus (MN) frequency was observed across all tested concentrations. Therefore, the *A. cepa* bioassays suggest that colistin sulphate exhibits mild toxicity, with potential cytotoxic and genotoxic effects under continuous exposure at 6.4 and 12.8 mg.L^-1^ concentrations. The maintenance of high MIC even after challenge with a suprainhibitory dose (6.4 mg·L ⁻ ¹) in cycle 11 reinforces the hypothesis that the acquired resistant phenotype does not depend on the external concentration of colistin, evidencing a stable and consolidated resistance profile.

## Conclusion

In multidrug-resistant Gram-negative infections, colistin remains a crucial last-resort antimicrobial [5]. However, our findings demonstrate that subinhibitory colistin exposure may contribute to the emergence of resistant strains. Notably, our data suggest a potential link between low-dose colistin exposure and the development of *mcr-1*-mediated resistance. We observed that such exposure induced a stable resistance phenotype in *E. coli* (ATCC 25922), leading to phenotypic expression consistent with *mcr-1* activity. The induced strain exhibited a different growth rate resembling clinical multidrug-resistant isolated and showed signs of collateral reduced susceptibility to ceftazidime. These findings support the hypothesis that *mcr-1*-mediated membrane modifications may confer selective advantages and impact susceptibility to other antimicrobials. Our results provide a general analysis of the consequences of sub-MIC exposure to colistin on the phenotypic characteristics of *E. coli*, using the standard strain *E. coli* (ATCC 25922). Furthermore, comparison with a positive control, the clinical isolate *E. coli* (CCBH 20178), offers insight into how the standard strain, following repeated exposure to sub-inhibitory concentrations of colistin, can exhibit phenotypic behaviors resembling those of a naturally resistant strain.

In addition, environmental toxicity assays revealed mild genotoxic and cytotoxic effects of colistin on *A. cepa*, suggesting ecological risks associated with its widespread use. Given the global significance *mcr-1* in resistance dissemination, investigations into factors promoting its emergence and spread are paramount. Furthermore, colistin showed mild toxicity and genotoxic and cytotoxic potential. Our results support the growing consensus that preserving colistin efficacy requires stringent antibiotic stewardship [5]. Colistin use should be restricted to cases without alternative treatments, as with all last-resort antimicrobials. This precautionary approach is essential to prevent widespread colistin resistance and maintain its therapeutic value for life-threatening infections.

## Supporting information

S1 File Supplementary tables 1 to 18. (ZIP)

## References

[1] Dadgostar P. Antimicrobial Resistance: Implications and Costs. Infect Drug Resist. 2019;12:3903–10. doi: 10.2147/IDR.S234610 31908502 PMC6929930

[2] Neill JO. Antimicrobial Resistance: Tackling a Crisis for the Health and Wealth of Nations. 2014.

[3] World Health Organization. Critically Important Antimicrobials for Human Medicine 6th Revision 2018 Ranking of Medically Important Antimicrobials for Risk Management of Antimicrobial Resistance Due to Non-Human Use. 2018.

[4] Of R C. Colistin: the revival of polymyxins for the management of multidrug-resistant gram-negative bacterial infections. Clin Infect Dis. 2005;40(9):1333. 15825037 10.1086/429323

[5] World Health Organization WHO. Global Antimicrobial Resistance Surveillance System (GLASS) The Detection and Reporting of Colistin Resistance. 2018.

[6] Sun J, Zhang H, Liu YH, Feng Y. Towards understanding MCR-like colistin resistance. Trends in Microbiology. 2018;26:794–808. 29525421 10.1016/j.tim.2018.02.006

[7] García V, García-Meniño I, Mora A, Flament-Simon SC, Díaz-Jiménez D, Blanco JE, et al. Co-occurrence of mcr-1, mcr-4 and mcr-5 genes in multidrug-resistant ST10 Enterotoxigenic and Shiga toxin-producing Escherichia coli in Spain (2006-2017). Int J Antimicrob Agents. 2018;52(1):104–8. doi: 10.1016/j.ijantimicag.2018.03.022 29635007

[8] Liu Y-Y, Wang Y, Walsh TR, Yi L-X, Zhang R, Spencer J, et al. Emergence of plasmid-mediated colistin resistance mechanism MCR-1 in animals and human beings in China: a microbiological and molecular biological study. Lancet Infect Dis. 2016;16(2):161–8. doi: 10.1016/S1473-3099(15)00424-7 26603172

[9] Gharaibeh MH, Shatnawi SQ. An overview of colistin resistance, mobilized colistin resistance genes dissemination, global responses, and the alternatives to colistin: A review. Vet World. 2019;12(11):1735–46. doi: 10.14202/vetworld.2019.1735-1746 32009752 PMC6925059

[10] Marciano DC, Wang C, Hsu T-K, Bourquard T, Atri B, Nehring RB, et al. Evolutionary action of mutations reveals antimicrobial resistance genes in Escherichia coli. Nat Commun. 2022;13(1):3189. doi: 10.1038/s41467-022-30889-1 35680894 PMC9184624

[11] Conceição-Neto OC, Aires CAM, Pereira NF, da Silva LHJ, Picão RC, Siqueira BN, et al. Detection of the plasmid-mediated mcr-1 gene in clinical KPC-2-producing Escherichia coli isolates in Brazil. Int J Antimicrob Agents. 2017;50(2):282–4. doi: 10.1016/j.ijantimicag.2017.05.003 28579456

[12] Ling Z, Yin W, Shen Z, Wang Y, Shen J, Walsh TR. Epidemiology of mobile colistin resistance genes mcr-1 to mcr-9. J Antimicrob Chemother. 2020;75(11):3087–95. doi: 10.1093/jac/dkaa205 32514524

[13] de Souza M, Sammarro Silva KJ, Garbuio M, Inada NM, Bagnato VS, Lima AR. Photon spectra effects tested on the vegetal model Allium cepa. J Biophotonics. 2023;16(12):e202300168. doi: 10.1002/jbio.202300168 37679880

[14] Brazilian Committee on Antimicrobial Susceptibility Testing. Tabela-pontos-de-corte-clinico-BrCAST-01-02-2025. 2025.

[15] Ching C, Zaman MH. Development and selection of low-level multi-drug resistance over an extended range of sub-inhibitory ciprofloxacin concentrations in Escherichia coli. Sci Rep. 2020;10(1):8754. doi: 10.1038/s41598-020-65602-z 32471975 PMC7260183

[16] Coppi M, Cannatelli A, Antonelli A, Baccani I, Di Pilato V, Sennati S, et al. A simple phenotypic method for screening of MCR-1-mediated colistin resistance. Clinical Microbiology and Infection. 2018;24(2):201.e1-201.e3. doi: 10.1016/j.cmi.2017.08.011 28827120

[17] Brazilian Committee on Antimicrobial Susceptibility Testing. Orientações do EUCAST para a detecção de mecanismos de resistência e resistências específicas de importância clínica e/ou epidemiológica versão 2.0. 2017.

[18] Brazilian Committee on Antimicrobial Susceptibility Testing. Tabela-pontos-de-corte-BrCAST-15-03-2023. 2023.

[19] Sullivan GJ, Delgado NN, Maharjan R, Cain AK. How antibiotics work together: molecular mechanisms behind combination therapy. Curr Opin Microbiol. 2020;57:31–40. doi: 10.1016/j.mib.2020.05.012 32619833

[20] Christaki E, Marcou M, Tofarides A. Antimicrobial Resistance in Bacteria: Mechanisms, Evolution, and Persistence. J Mol Evol. 2020;88(1):26–40. doi: 10.1007/s00239-019-09914-3 31659373

[21] Li B, Yin F, Zhao X, Guo Y, Wang W, Wang P, et al. Colistin Resistance Gene mcr-1 Mediates Cell Permeability and Resistance to Hydrophobic Antibiotics. Front Microbiol. 2020;10:3015. doi: 10.3389/fmicb.2019.03015 31998280 PMC6966882

[22] Yang Q, Li M, Spiller OB, Andrey DO, Hinchliffe P, Li H, et al. Balancing mcr-1 expression and bacterial survival is a delicate equilibrium between essential cellular defence mechanisms. Nature Communications. 2017;8(1). 10.1038/s41467-017-02149-0 PMC5727292 29233990

[23] Ma W, Jiang X, Dou Y, Zhang Z, Li J, Yuan B, et al. Biophysical Impact of Lipid A Modification Caused by Mobile Colistin Resistance Gene on Bacterial Outer Membranes. J Phys Chem Lett. 2021;12(48):11629–35. doi: 10.1021/acs.jpclett.1c03295 34817187

[24] Esposito F, Fernandes MR, Lopes R, Muñoz M, Sabino CP, Cunha MP, et al. Detection of colistin-resistant mcr-1-positive Escherichia coli by use of assays based on inhibition by EDTA and zeta potential. J Clin Microbiol. 2017;55(12):3454–65. 28978685 10.1128/JCM.00835-17 PMC5703812

