# Supplementary material for: Induction of colistin resistance and environmental toxicity assessment in Escherichia coli
Source: PLoS One. 2026 Apr 21;21(4):e0340467. doi: 10.1371/journal.pone.0340467 (PMC13098942; doi:10.1371/journal.pone.0340467)
Supplement: S1 File — (ZIP) [file pone.0340467.s001.zip › Files/S1. Table 1.MIC of E coli (ATCC 25922) during sub-MIC concentrations exposed..pdf]

| *E. coli* ATCC (25922) | MIC* (mg/L) | Absolute deviation from the median |
|---|---|---|
| **Cycle 0** | 2 | 0 |
| **Cycle 2** | 2 | 0 |
| **Cycle 4** | 2 | 0 |
| **Cycle 6** | 2 | 0 |
| **Cycle 8** | 2 | 0 |
| **Cycle 10** | 2 | 0 |
| **Cycle 11** | 2 | 0 |

*: median
