# Supplementary material for: Induction of colistin resistance and environmental toxicity assessment in Escherichia coli
Source: PLoS One. 2026 Apr 21;21(4):e0340467. doi: 10.1371/journal.pone.0340467 (PMC13098942; doi:10.1371/journal.pone.0340467)
Supplement: S1 File — (ZIP) [file pone.0340467.s001.zip › Files/S1. Table 12. Bacterial growth curve for the E. coli strains.pdf]

Concentration (mg/L)	Germination index*	Standard deviation
**0**	67.77778	1.11111
**1.1**	34.44444	4.84322
**6.1**	34.44444	2.22222
**12.8**	27.77778	2.22222

*: mean
