# Supplementary material for: Induction of colistin resistance and environmental toxicity assessment in Escherichia coli
Source: PLoS One. 2026 Apr 21;21(4):e0340467. doi: 10.1371/journal.pone.0340467 (PMC13098942; doi:10.1371/journal.pone.0340467)
Supplement: S1 File — (ZIP) [file pone.0340467.s001.zip › Files/S1. Table 14. Germination index of A. cepa seeds after exposed of colistin.pdf]

Concentration (mg/L)	Root elongation*	Standard deviation
**0**	10.52951	0.22335
**1.1**	9.83656	1.01185
**6.1**	10.70997	1.4521
**12.8**	10.23825	0.94776

*: mean
