# Supplementary material for: Induction of colistin resistance and environmental toxicity assessment in Escherichia coli
Source: PLoS One. 2026 Apr 21;21(4):e0340467. doi: 10.1371/journal.pone.0340467 (PMC13098942; doi:10.1371/journal.pone.0340467)
Supplement: S1 File — (ZIP) [file pone.0340467.s001.zip › Files/S1. Table 16. Mitotic index of meristematic cell of A. cepa seeds after exposed of colistin.pdf]

**Concentration (mg/L)**	**Chromosomal aberrations***	**Standard deviation**
**0**	0	0
**1.1**	0,19875	0,05415
**6.1**	0,42692	0,04878
**12.8**	0,64116	0,09471

*: mean
