# Supplementary material for: Induction of colistin resistance and environmental toxicity assessment in Escherichia coli
Source: PLoS One. 2026 Apr 21;21(4):e0340467. doi: 10.1371/journal.pone.0340467 (PMC13098942; doi:10.1371/journal.pone.0340467)
Supplement: S1 File — (ZIP) [file pone.0340467.s001.zip › Files/S1. Table 17. Chromosomal aberrations index of meristematic cell of A. cepa seeds after exposed of colistin.pdf]

**Concentration (mg/L)**	**Micronuclei ***	**Absolute deviation from the median**
**0**	0	0
**1.1**	0.06208	0.02006
**6.1**	0.07076	0.02341
**12.8**	0.09403	0.01429

*: mean
