# Supplementary material for: Induction of colistin resistance and environmental toxicity assessment in Escherichia coli
Source: PLoS One. 2026 Apr 21;21(4):e0340467. doi: 10.1371/journal.pone.0340467 (PMC13098942; doi:10.1371/journal.pone.0340467)
Supplement: S1 File — (ZIP) [file pone.0340467.s001.zip › Files/S1. Table 18. Micronuclei index of meristematic cell of A. cepa seeds after exposed of colistin.pdf]

Cycle 1

Strain	MIC CAMHB* (mg/L)	absolute deviation from the median	MIC CAMHB*,# (mg/L)	absolute deviation from the median
E. coli (ATCC 25922)	2	0	2	0.5
E. coli (C11)	8	0	16	0
E. coli (CCBH 20178)	8	0	16	0

Cycle 5

Strain	MIC – CAMHB* (mg/L)	absolute deviation from the median	MIC CAMHB*,# (mg/L)	absolute deviation from the median
E. coli (ATCC 25922)	2	0.5	2	1
E. coli (C11)	8	0	32	0
E. coli (CCBH 20178)	8	0	32	0

*: median

#: calcium chloride-enriched MHCA (5 mM)

MHCAB: Mueller Hinton cations adjusted broth
