# Supplementary material for: Induction of colistin resistance and environmental toxicity assessment in Escherichia coli
Source: PLoS One. 2026 Apr 21;21(4):e0340467. doi: 10.1371/journal.pone.0340467 (PMC13098942; doi:10.1371/journal.pone.0340467)
Supplement: S1 File — (ZIP) [file pone.0340467.s001.zip › Files/S1. Table 3. Colistin MIC of E. coli (C 11) during six free antibiotic growth cycles.pdf]

| *E. coli* (ATCC 25922) | **MIC* (mg/L)** | **Absolute deviation from the median** |
|---|---|---|
| **Cycle 2** | 1 | 0 |
| **Cycle 4** | 1 | 0 |
| **Cycle 6** | 1 | 0 |

*: median
