# Supplementary material for: Induction of colistin resistance and environmental toxicity assessment in Escherichia coli
Source: PLoS One. 2026 Apr 21;21(4):e0340467. doi: 10.1371/journal.pone.0340467 (PMC13098942; doi:10.1371/journal.pone.0340467)
Supplement: S1 File — (ZIP) [file pone.0340467.s001.zip › Files/S1. Table 7. Ceftazidime MIC of E. coli (ATCC 25922) during six free antibiotic growth cycles.pdf]

***E. coli* (CCBH 20178)**	**MIC* (mg/L)**	**Absolute deviation from the median**
**Cycle 2**	16	0
**Cycle 4**	16	0
**Cycle 6**	16	0

*: median
