# Supplementary material for: Induction of colistin resistance and environmental toxicity assessment in Escherichia coli
Source: PLoS One. 2026 Apr 21;21(4):e0340467. doi: 10.1371/journal.pone.0340467 (PMC13098942; doi:10.1371/journal.pone.0340467)
Supplement: S1 File — (ZIP) [file pone.0340467.s001.zip › Files/S1. Table 9. Gentamicin MIC of E. coli (C 11) during six free antibiotic growth cycles.pdf]

**Time**	***E. coli* (ATCC 22922)***	**Standard deviation**	***E. coli* (C 11)***	**Standard deviation**	***E. coli* (CCBH 20178)***	**Standard deviation**
**0**	0.00127	2,33E+01	0.00896	0.00579	-0.00225	9,88E+00
**900.29**	0.0012	2,65E+01	0.00563	0.00501	-0.00225	0.00127
**1800.29**	0.00137	3,48E+00	0.00536	0.00496	-0.00225	0.00104
**2700.29**	0.0014	5,51E+01	0.00538	0.005	-0.00127	8,77E+01
**3600.29**	0.00123	3,18E+00	0.00466	0.00442	-0.00127	6,27E+01
**4500.29**	0.0015	3,06E+01	0.00539	0.00504	-0.0016	5,54E+01
**5400.29**	0.00103	3,53E+01	0.00483	0.00445	-0.00157	6,29E+01
**6300.29**	0.00133	2,91E+01	0.0056	0.00474	-5.5E-4	7,12E+01
**7200.29**	0.0015	3,21E+01	0.00667	0.00512	-6.5E-4	7,69E+01
**8100.29**	0.00233	5,61E+01	0.00771	0.00517	4,83E+01	0.00104
**9000.29**	0.00243	4,18E+01	0.01017	0.00536	0.00217	0.00142
**9900.29**	0.00367	5,78E+01	0.01437	0.00581	0.00583	0.00238
**10800.29**	0.00593	0.0013	0.0216	0.00659	0.01287	0.00419
**11700.29**	0.0103	0.00214	0.03443	0.00813	0.02513	0.00739
**12600.29**	0.0179	0.00418	0.05453	0.01055	0.04433	0.01126
**13500.29**	0.0324	0.00683	0.08189	0.01324	0.07048	0.01633
**14400.29**	0.05377	0.00875	0.11718	0.01605	0.10397	0.02183
**15300.29**	0.07863	0.01004	0.16234	0.01894	0.14975	0.02863
**16200.29**	0.1081	0.0126	0.2165	0.02152	0.20397	0.03508

**17100.29**	0.1481	0.0191	0.27393	0.02307	0.2625	0.03691
**18000.29**	0.21133	0.02614	0.32919	0.02361	0.32045	0.03308
**18900.29**	0.29333	0.02895	0.37951	0.02319	0.36763	0.03212
**19800.29**	0.38063	0.02984	0.42967	0.02208	0.39597	0.03231
**20700.29**	0.46847	0.02311	0.46814	0.01987	0.42173	0.0314
**21600.29**	0.51837	0.01054	0.49896	0.01749	0.44182	0.02884
**22500.29**	0.52097	0.00997	0.52543	0.01536	0.45542	0.0268
**23400.29**	0.51597	0.00908	0.54673	0.01401	0.4664	0.02602
**24300.29**	0.5237	0.00578	0.56614	0.01367	0.47442	0.02524
**25200.29**	0.53613	0.00344	0.58371	0.01344	0.48193	0.02519
**26100.29**	0.5456	0.00223	0.59887	0.01309	0.49235	0.02425
**27000.29**	0.5519	0.00155	0.61281	0.01254	0.49998	0.02377
**27900.29**	0.5567	3,61E+01	0.62696	0.01217	0.50873	0.02388
**28800.29**	0.56087	7,88E+01	0.64022	0.01179	0.51728	0.02394
**29700.29**	0.56537	0.00129	0.65298	0.01153	0.52908	0.02544
**30600.29**	0.57037	0.00212	0.66497	0.01155	0.54032	0.02742
**31500.29**	0.57577	0.00285	0.67611	0.01151	0.5488	0.02842
**32400.29**	0.58043	0.00283	0.68667	0.0116	0.55767	0.02946
**33300.29**	0.58503	0.0037	0.69653	0.0115	0.56688	0.02937
**34200.29**	0.58973	0.00421	0.70651	0.0118	0.57625	0.0291
**35100.29**	0.59463	0.00491	0.71559	0.01207	0.58527	0.02937
**36000.29**	0.60067	0.00564	0.72468	0.01252	0.59302	0.02953
**36900.29**	0.60767	0.0062	0.73363	0.01302	0.60108	0.02979
**37800.29**	0.617	0.00752	0.74253	0.01337	0.6081	0.03021

*: mean of optical density of growth at 37°C
